# The human hippocampus and its subfield volumes across age, sex and APOE e4 status

**DOI:** 10.1093/braincomms/fcaa219

**Published:** 2020-12-19

**Authors:** Michele Veldsman, Lisa Nobis, Fidel Alfaro-Almagro, Sanjay Manohar, Masud Husain

**Affiliations:** 1 Wellcome Centre for Integrative Neuroimaging, University of Oxford, Oxford, UK; 2 Department of Experimental Psychology, University of Oxford, Oxford, UK; 3 Oxford Centre for Human Brain Activity, Wellcome Centre for Integrative Neuroimaging, Department of Psychiatry, University of Oxford, Oxford, UK; 4 Nuffield Department of Clinical Neuroscience, John Radcliffe Hospital, University of Oxford, Oxford, UK; 5 Division of Clinical Neurology, John Radcliffe Hospital, Oxford University Hospitals Trust, Oxford, UK

**Keywords:** hippocampus, Alzheimer’s disease, ageing, subfields

## Abstract

Female sex, age and carriage of the apolipoprotein E e4 allele are the greatest risk factors for sporadic Alzheimer’s disease. The hippocampus has a selective vulnerability to atrophy in ageing that may be accelerated in Alzheimer’s disease, including in those with increased genetic risk of the disease, years before onset. Within the hippocampal complex, subfields represent cytoarchitectonic and connectivity based divisions. Variation in global hippocampal and subfield volume associated with sex, age and apolipoprotein E e4 status has the potential to provide a sensitive biomarker of future vulnerability to Alzheimer’s disease. Here, we examined non-linear age, sex and apolipoprotein E effects, and their interactions, on hippocampal and subfield volumes across several decades spanning mid-life to old age in 36 653 healthy ageing individuals. FMRIB Software Library derived estimates of total hippocampal volume and Freesurfer derived estimates hippocampal subfield volume were estimated. A model-free, sliding-window approach was implemented that does not assume a linear relationship between age and subfield volume. The annualized percentage of subfield volume change was calculated to investigate associations with age, sex and apolipoprotein E e4 homozygosity. Hippocampal volume showed a marked reduction in apolipoprotein E e4/e4 female carriers after age 65. Volume was lower in homozygous e4 individuals in specific subfields including the presubiculum, subiculum head, cornu ammonis 1 body, cornu ammonis 3 head and cornu ammonis 4. Nearby brain structures in medial temporal and subcortical regions did not show the same age, sex and apolipoprotein E interactions, suggesting selective vulnerability of the hippocampus and its subfields. The findings demonstrate that in healthy ageing, two factors—female sex and apolipoprotein E e4 status—confer selective vulnerability of specific hippocampal subfields to volume loss.

## Teaser

Female sex, age and carriage of the APOE e4 allele are the greatest risk factors for sporadic Alzheimer's disease. Analysis of these three risk factors on hippocampal and subfield volumes in 36 653 healthy ageing individuals show two factors—female sex and APOE e4 status—confer selective vulnerability of specific hippocampal subfields to volume loss.

## Graphical Abstract

**Graphical Abstract:**
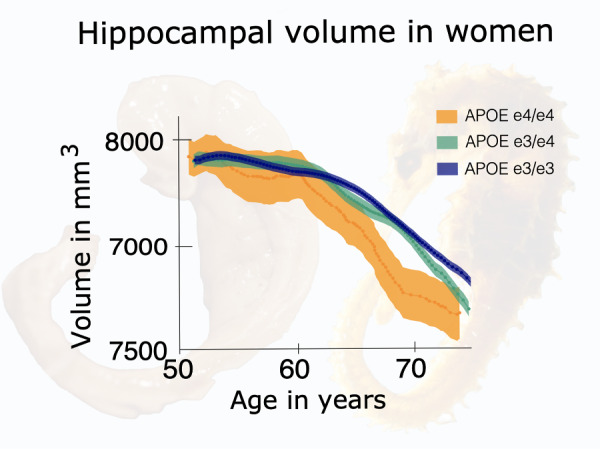

## Introduction

The human hippocampus has a long-established role in episodic and long-term memory (Eichenbaum *et al.*, 2012; Moscovitch *et al.*, 2016). More recently, studies in healthy individuals, patients with hippocampal damage and those at risk of Alzheimer’s disease indicate a role for the hippocampus also in short-term and working memory (Hartley *et al.*, 2007; Yonelinas, 2013; Liang *et al.*, 2016; Zokaei and Husain, 2019). Given the central role of the hippocampus in memory, it is not surprising that it is also implicated in Alzheimer’s disease (Moodley and Chan, 2014), whose typical presentation is progressively severe memory impairment (McKhann *et al.*, 2011). Age, female sex and carriage of the apolipoprotein E (APOE)-e4 allele are the greatest risk factors for sporadic Alzheimer’s disease (Riedel *et al.*, 2016) and their association with hippocampal volume is therefore of great interest.

Meta-analyses of brain structure confirm that there are significant regional volume and tissue density differences between males and females in the hippocampus across the adult lifespan (Ruigrok *et al.*, 2014). Sex interacts with age, such that hippocampal volume differences are more pronounced in later life. Indeed, a recent analysis of 19 793 healthy individuals (mean age 62.95, SD 7.48) demonstrated accelerated hippocampal volume loss at middle age that was more prominent in females than males (Nobis *et al.*, 2019). To complicate matters, decreases in brain volumes with advancing age, including of the hippocampus, appear to be non-linear with a relatively steep decline in later life (Ziegler *et al.*, 2012). Alzheimer’s disease is associated with hippocampal atrophy, with volume loss significantly greater than observed in healthy aging (Jack *et al.*, 2000; Wang *et al.*, 2003; Schuff *et al.*, 2008). Age remains the strongest predictor of Alzheimer’s disease and the same processes that have been identified to drive ageing are also major risk factors for Alzheimer’s disease. Glucose hypometabolism, mitochondrial dysfunction, inflammatory and immune responses, beta-amyloid processing and white matter degeneration all produce an environment susceptible to the development of Alzheimer’s disease pathology (Riedel *et al.*, 2016).

The hippocampus can be partitioned into subfields based on cellular architecture and connectivity (Iglesias *et al.*, 2015). The broadest subfield divisions include the subiculum, the dentate gyrus and the cornu ammonis (subdivided into CA1, CA2, CA3, CA4). Advances in magnetic resonance imaging (MRI) spatial resolution and automated segmentation algorithms using ultra-high-resolution *exvivo* MRI reference templates (Iglesias *et al.*, 2015), have allowed increasingly precise estimation of the volume of the subfields. It is now evident that subfields are differentially impacted across neurological diseases (Nadal *et al.*, 2020) suggesting selective vulnerabilities to different pathologies with varying phenotypic outcomes. Examination of the effects of age across the entire hippocampus may mask non-linear trajectories that reflect some of the unique properties of hippocampal subfields. Post-mortem studies provide evidence of neuronal loss of the dentate gyrus, subiculum and CA1 in the range of 32–67% across the lifespan (West *et al.*, 1994; Simić *et al.*, 1997). Voxel-based morphometry analysis of structural MRI has sometimes agreed with this, finding reduced volume in the subiculum in ageing (Chételat *et al.*, 2008; La Joie *et al.*, 2010; Thomann *et al.*, 2013) but the subfields implicated vary widely across studies (Olsen *et al.*, 2019; Nadal *et al.*, 2020).

There is some evidence of CA1 and the subiculum being the first subfields implicated at the earliest stages of Alzheimer’s disease before the entire hippocampus degenerates in the course of the disease (Mueller and Weiner, 2009; de Flores *et al.*, 2015). APOE is a critical cholesterol and triglyceride transporter necessary for the maintenance of neuronal membranes and myelin, with APOE-e4 allele carriers being at significantly higher risk of developing Alzheimer’s disease (Riedel *et al.*, 2016). In fact, it represents the biggest genetic risk factor for sporadic Alzheimer’s disease in healthy ageing (Farrer *et al.*, 1997). Furthermore, age and APOE e4 carriage interact with sex: female e4 heterozygotes have an increased risk of Alzheimer’s disease 5 years earlier than non-carriers (Noguchi *et al.*, 1993). Fewer studies have examined the association between APOE e4 status on hippocampal subfield volume than the effect of Alzheimer’s disease itself. Where it has been investigated, there are wide variations in the subfields affected and those that are specifically associated with APOE e4 status as opposed to general ageing (de Flores *et al.*, 2015; Nadal *et al.*, 2020).

The disagreement between reports is due to small sample sizes (particularly regarding the rare APOE e4/e4 variant), varied subfield segmentation methods and classifications, and different age spans studied. Here, we aim to provide more definitive insights into the impact of Alzheimer’s disease risk factors on hippocampal complex volumes, making several advances in the current literature. First, we take advantage of the large UK Biobank sample to provide the biggest sample of APOE e4 positive individuals studied for this purpose. Second, we use population data to examine trajectories of hippocampal subfield volumes spanning several decades from mid-life to old age, for each sex. In addition, we use a model-free sliding-window approach that does not make linear assumptions on the relationship between age and subfield volume. Our hypothesis is that hippocampal subfields will differ in their relative preservation, versus decline, in volume from mid to late life, showing a steeper trajectory associated with Alzheimer’s disease risk factors of female sex, older age and APOE e4 positive status.

## Materials and methods

### Participants

**Figure 1:**
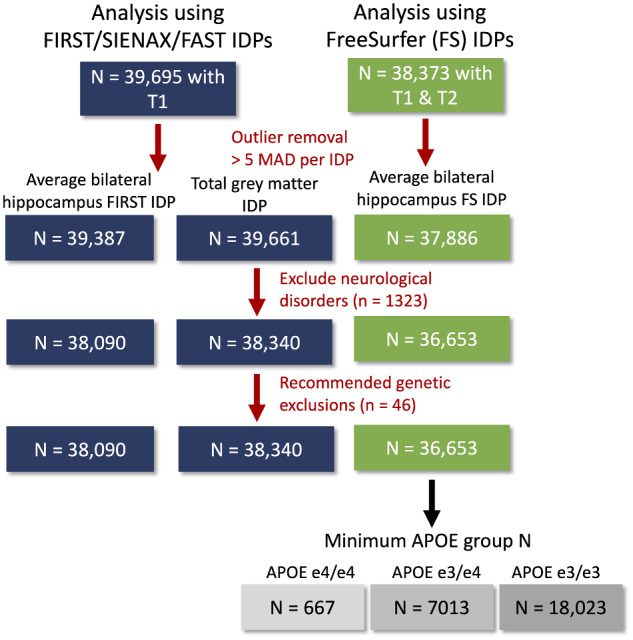
**Flowchart for exclusions.** Fewer participants had available data on FreeSurfer (FS) IDPs, because presence of both T1 and T2-weighted images were required. Outliers were removed for each IDP separately. Finally, participants with a neurological or psychiatric disorder or genetic data not meeting quality control were excluded from all IDP data.

Data from 39 695 participants from the UK Biobank were analysed, the maximum number of participants with imaging data available at the time of analysis. Participants self-reporting a history or current diagnosis of neurological or psychiatric disorder, head injury or substance abuse were excluded from analysis (see Fig. 1 for exclusions).

Genotyping was conducted by Affymetrix for UK Biobank using bespoke Axiom arrays. Full details of the genotyping pipeline are openly available (Bycroft *et al.*, 2018). As we were interested in hippocampal volume differences, we focused on heterozygous and homozygous e4 carriers only as they have been repeatedly shown to have reduced hippocampal volume to non-carriers and e2 carriers (Geroldi *et al.*, 1999; Agosta *et al.*, 2009).

### Image acquisition and analysis

Brain images were acquired on a Siemen’s Skyra 3 T scanner with a 32-channel head coil (Siemen’s Medical Solutions, Germany). High-resolution (1 mm isotropic voxel), T1-weighted, 3D magnetization-prepared gradient echo structural images and a T2 weighted fluid-attenuated inversion recovery (FLAIR) images (1.05 mm × 1 mm × 1 mm resolution) were acquired as part of a longer MRI protocol, full details of which are openly available here. Preprocessing and quality checking of images followed a standardized and openly available pipeline (https://www.fmrib.ox.ac.uk/ukbiobank/protocol/index.html) the details of which have been published elsewhere (Miller *et al.*, 2016; Alfaro-Almagro *et al.*, 2018). Our analysis was based on imaging derived phenotypes (IDPs), summary statistics representing key brain imaging variables, in this case, Freesurfer estimated hippocampal subfield volumes and total grey matter, total hippocampal and medial temporal lobe volume IDPs estimated in FMRIB software library (Jenkinson *et al.*, 2012).

**Figure 2:**
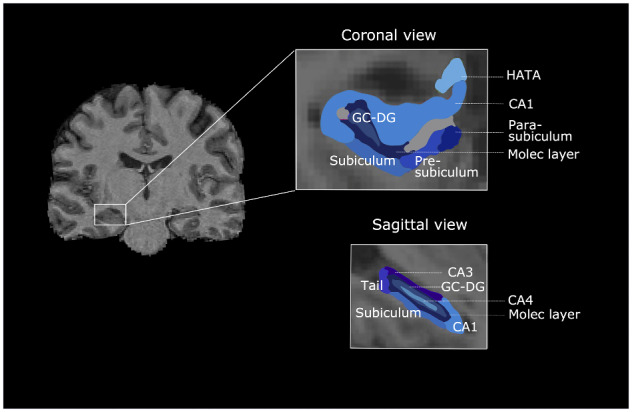
**Freesurfer parcellation of hippocampal subfields in coronal and sagittal views.** CA1–4 = cornu ammonis; HATA = hippocampus-amygdala-transition-area; Molec layer = molecular layer; GC-DC = granule cell layer of dentate gyrus.

For the subfield volume estimates, the T1 and T2 fluid-attenuated inversion recovery images were input into Freesurfer 6.0 (https://surfer.nmr.mgh.harvard.edu/) to estimate subcortical volumes. Freesurfer 6.0 segments the hippocampus into 13 segments using a probabilistic, high-resolution *exvivo* atlas based on 15 subjects scanned at 7 T (∼0.1 mm isotropic). The *exvivo* atlas has an isotropic resolution of 0.13 mm making it possible to delineate the subfields to a high degree of accuracy (Iglesias *et al.*, 2015). The 13 regions are detailed in Iglesias *et al.* (2015) and presented in Fig. 2.

### Data preprocessing

We used the median absolute deviation to identify and exclude outliers. An absolute deviation of greater than five from the median was chosen as a cut-off based on visual inspection of scatterplots of age × subfield volume. The median absolute deviation is robust to deviations from normality in variables. The variance associated with confounding variables were regressed from IDPs. Confounds included scanning date, table position and head size (Alfaro-Almagro *et al.*, 2018). Head size is a scaling factor based on the transformation of the individuals structural MRI to the standard template space. This confound is particularly important in the investigation of sex effects of brain volume since many brain structure volumes scale with head size (Miller *et al.*, 2016). By correcting for these confounds, our results are independent from individual differences in scanning date, table position and head size.

### Statistical analysis

Jamovi (The jamovi project, 2020) was used to perform analyses of variance. All other calculations were done in MATLAB 2019b (The MathWorks, Inc., 2019).

We first examined the trajectory of whole bilateral hippocampal volume, and hippocampal subfields, across age in heterozygous (e3/e4) and homozygous (e4/e4) carriers compared to homozygous e3 carriers (e3/e3). We did the same analysis splitting by sex, using sliding-window curves (described below). We plotted the trajectories of neighbouring medial temporal regions including the superior temporal gyrus, middle temporal gyrus, inferior temporal gyrus, fusiform gyrus, parahippocampal gyrus and temporal pole to examine hippocampal specific APOE effects across age.

In addition to sliding-window curves, to formally assess the interaction between age, sex and APOE status, two-way analyses of variance tested for significant APOE and sex effects, splitting the data into two age groups (<65 and ≥65). The cut-off age was chosen based on sliding-window results. We calculated a full factorial model, with APOE group (e3/3, e3/4, e4/4 and a group with all remaining participants who were not members of the three other groups, ‘neither’), age group and sex as fixed factors, and either bilateral hippocampus, or total grey matter volume, as dependent variables.

To quantify the different effects of sex and APOE status on the annual cross-sectional change in volume, we calculated the slope of age, using reduced linear regression models for each fixed effect: (i) volume ∼ age; (ii) volume ∼ age + age * sex; and (iii) volume ∼ age + age*APOE. This was done separately for all hippocampal subfields.

### Sliding-window curves

Model-free sliding-window curves were implemented using a fixed-width age-quantile window moved along the age distribution (code available here: https://osf.io/vmabg/; conditionalPlot.m).

Each window contained 10% of the participants, overlapping with the previous and/or following window. Mean volumes for the hippocampus and its subfields were calculated within each window. The mean volumes in each window were then smoothed with Gaussian kernel of width 20%. This smoothing kernel was chosen based on previous work using this method (Nobis *et al.*, 2019). We plot the smoothed mean volumes as a function of age for each volume of interest, with standard errors of the mean. Note that because there are more data points around the mean age, and fewer data points at extreme ages, the lowest and highest percentile windows include a wider age range.

### Permutation testing on sliding-window curves

Differences between mean volumes per window for APOE e3/e3 and e4/e4 carriers were tested using a permuted t-statistic, correcting *P*-values for false discovery rate. This was done by shuffling data points for each matching age window of the two different APOE groups. We then tested 5000 permuted datasets per window and calculated the *P*-value as the proportion of permutations that resulted in a t-statistic for a difference between the volume means that was as least as extreme as the one observed in the original data.

This method relies on the assumption of matching age distributions, which, based on non-significant Kolmogorov–Smirnov (KS) tests, was met. As the KS test is more likely to return significant results with large sample sizes, we computed a cut-off threshold and confidence interval for the KS statistic based on bootstrapped samples from the distributions. For example, to determine whether the age distributions of the APOE e3/e3 and APOE e4/e4 carrier groups are significantly different from each other, we took 5000 bootstrapped samples of each of them. Calculating the result of the KS test (D statistic) for the differences between the original APOE e3/e3 and each of its bootstrapped samples, as well as between the original APOE e4/e4 and each bootstrapped sample, resulted in an approximation of the null distribution for each of the two groups. We then calculated the width of each null distribution by subtracting the value of the first and 99th percentile and divided the result by two. To get a cut-off threshold for determining significance, the width of each distribution was added to the maximum of their means. Finally, we computed a confidence interval for the D statistic from the KS test on the difference between the APOE e3/e3 and APOE e4/e4 groups. This was done by first calculating the D statistic for the differences between the original APOE e3/e3 distribution and each of the bootstrapped APOE e4/e4 samples, as well as between the original APOE e4/e4 distribution and each of the bootstrapped APOE e3/e3 samples. The 99% confidence interval for the alternative hypothesis that the two groups are different is then based on the first and 99th percentile values of the resulting D statistics. Thus, if this confidence interval includes the cut-off threshold, the age distributions are not significantly different with 99% confidence.

## Data availability

We used data from the UK Biobank which can be requested by any researcher on application (https://www.ukbiobank.ac.uk). Our sliding-window analysis script is available openly here: (https://osf.io/vmabg/).

## Results

### Demographics

Participants with complete data on APOE status were between 44 and 82 years old. Table 1 shows mean age with standard deviation by sex and APOE status, as well as the number of participants with lower and higher education levels. There was a significant difference in mean age between the APOE groups, but with a very small effect size. *Post hoc* analysis showed a significant difference in mean age between APOE e3/e4 and APOE e3/e3 carriers (*P* < 0.001, Cohen’s D = 0.000006), as well as between APOE e3/e4 carriers and people with neither of these three combinations (*P* < 0.001, Cohen’s D = 0.000039), again with negligible effect size. A detailed analysis of sociodemographic characteristics of the entire UK Biobank cohort describes its generalisability to the general population (Fry *et al.*, 2017).

**Table 1:** Mean age and standard deviation by sex and APOE status

	Women	Men	All	ALL
	Age in years	Age in years	Age in years	Education level
	(mean ± std)	(mean ± std)	(mean ± std)	(no. of lower/higher)
APOE e3/e3	63.59 ± 7.42	64.0 ± 7.67	64.16 ± 7.56	7058/12461
APOE e3/e4	63.2 ± 7.32	63.55 ± 7.69	63.78 ± 7.52	2760/4865
APOE e4/e4	62.53 ± 7.07	62.54 ± 7.47	63.53 ± 7.33	231/493
Other	63.58 ± 7.43	63.68 ± 7.56	64.29 ± 7.53	357/602
*P*			F(3,39646) = 9, *P* < 0.001	
Eta squared			<0.01	

### Selective vulnerability of the hippocampus to age, sex and APOE status

**Figure 3:**
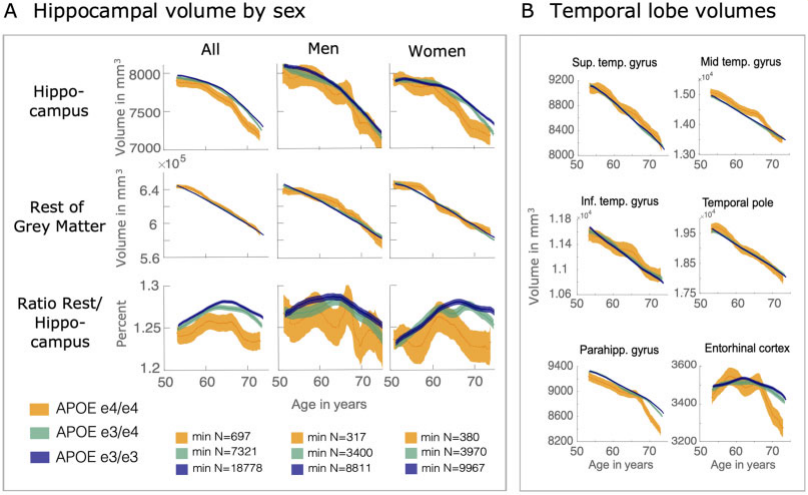
**Accelerated hippocampal volume loss in APOE e4/e4 carriers in older age.** (**A**) Hippocampal volume across age and divided by APOE group, in the whole sample, and for women and men separately. The acceleration of hippocampal volume loss that is increased in APOE e4/e4 carriers becomes clear when comparing hippocampal volume with total grey matter loss. The ratio of hippocampal volume to the rest of grey matter shows increased hippocampal volume loss relative to the rest of grey matter when its slope turns negative. (**B**) Temporal lobe volumes decrease largely linear across age for all APOE groups, with the exception of parahippocampal gyrus volume, which shows a similar trajectory to the hippocampus.

First, bilateral hippocampal and other medial temporal volumes were examined in APOE e3/e3, e3/e4 and e4/e4 carriers, using a model-free sliding-window approach. Bilateral hippocampal volume was reduced in APOE e4/e4 carriers compared to e3/3 and e3/e4 carriers. This effect was especially clear when examining the ratio of hippocampal volume to the rest of the grey matter, across age (Fig. 3A), suggesting selective vulnerability of the entire hippocampus. Brain volume reduced with age across all other medial temporal lobe regions, but with no clear effect of APOE status (Fig. 3B), except for the parahippocampal gyrus and ento.

For all APOE groups there was reduced hippocampal volume relative to total grey matter volume from age ∼65, which was most pronounced in e4/e4 carriers. Furthermore, this trajectory appeared to be driven by sex, with female e4/e4 carriers exhibiting a markedly lower hippocampal volume from age 65 onwards (Fig. 3A). Again, this was more prominent when calculated as the ratio of hippocampal volume to total grey matter volume (Fig. 3A).

**Figure 4:**
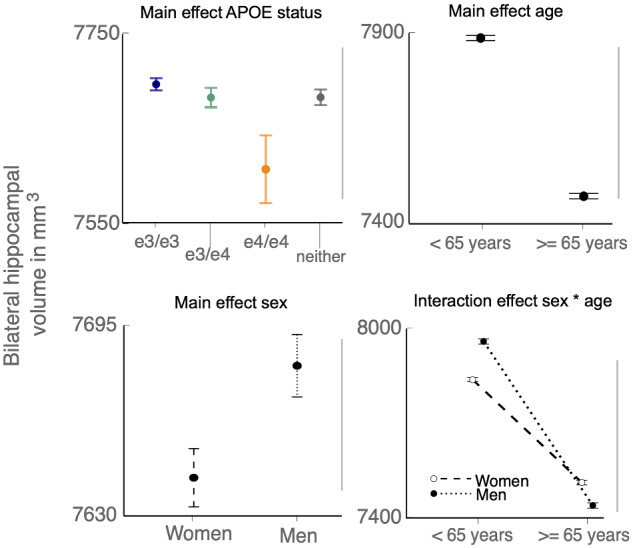
**Replication of main effects and sex-by-age interaction with analyses of variance.** Significant linear main effects were found for APOE status, age, and sex, as well as a sex-by-age interaction, but not for interactions with APOE status.

To quantify the relationship between sex, APOE status and total bilateral hippocampal volume compared to total grey matter volume and their interactions, we conducted between groups analyses of variance for e3/e3 and e3/e4 compared to e4/e4 carriers split by age group (<65 and ≥65). With total bilateral hippocampal volume as the dependent variable, there were significant effects of APOE status [*F*(3,37864) = 5.53, *P* < 0.001], age [*F*(1,37864) = 821.67, *P* < 0.001], sex [*F*(1,37864) = 6.85, *P* < 0.05], as well as a sex-by-age interaction [*F*(1,37864) = 21.58, *P* < 0.001] (Fig. 4).

In contrast, only age had a significant main effect on total grey matter volume [*F*(1,38282) = 3401.53, *P* < 0.001], with no significant interactions between APOE status, sex and age [*F*(3,38282) = 0.82, *P* = 0.483]. The results confirm a selective vulnerability of the hippocampus compared to the rest of the grey matter to sex and APOE status.

### Interactions between age and APOE status on selective hippocampal subfield volumes

**Figure 5:**
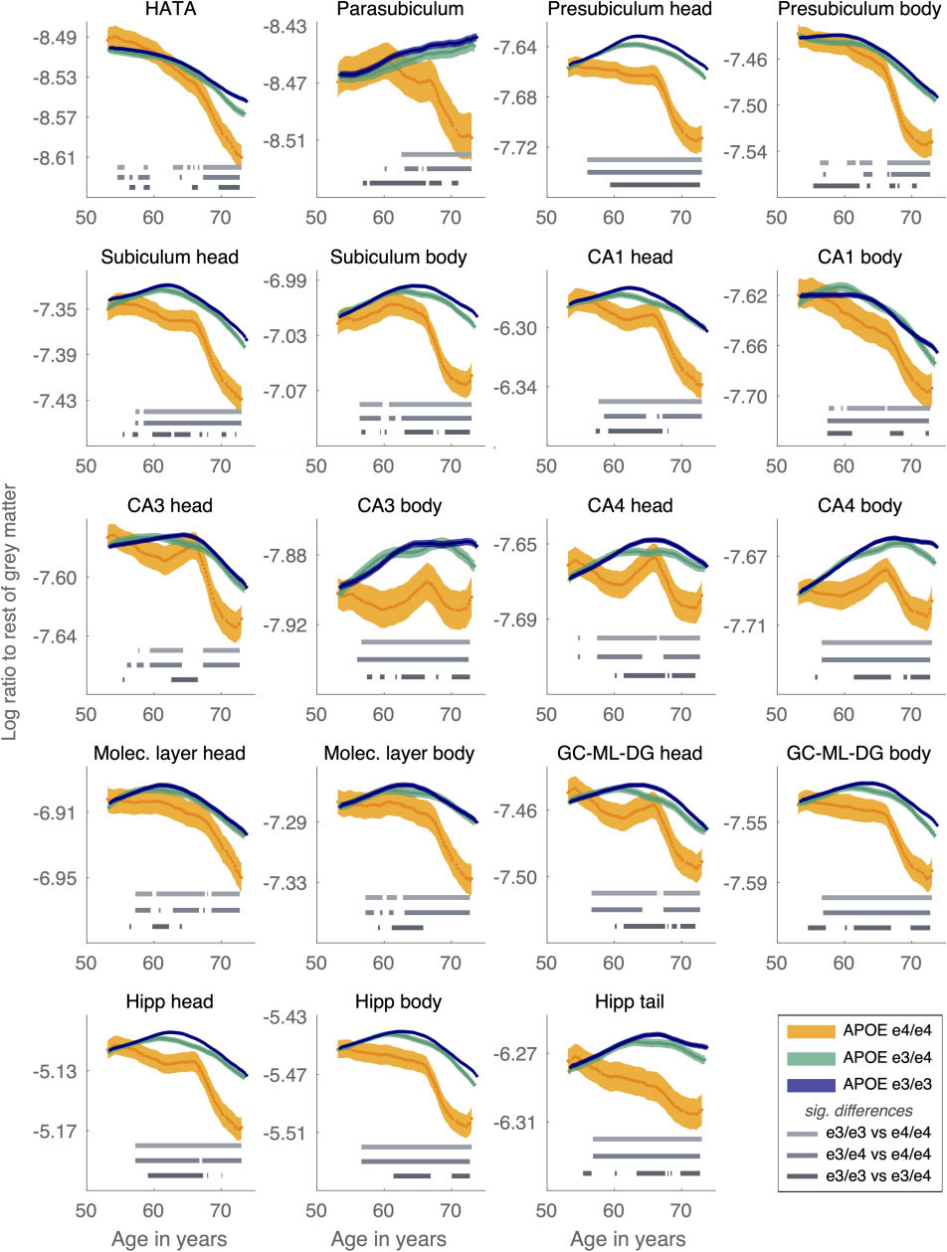
**Ratio of hippocampal subfields to the rest of grey matter.** A negative slope of the log ratio indicates larger volume loss relative to the rest of grey matter. For many subfields the slope becomes negative around age 65 years, which is especially pronounced in APOE e4/e4 carriers. Significant differences between the curves at *P* < 0.001 are shown per age window as grey horizontal bars.

Next, hippocampal subfield volume, split by APOE status, was examined as the log ratio of subfield volume to total grey matter volume across age (Fig. 5), to ensure subfield trajectories accounted for global brain volume loss in ageing. Differences between the mean volumes per age bin, across APOE e3 and e4 carriers were tested using a permuted t-statistic (FDR corrected). The plotted trajectories of subfield volumes independent of total grey matter volume are presented in Supplementary Fig. 1 and show the same pattern of results.

In all subfields, APOE e3/e3 and e3/e4 carriers had highly similar and overlapping trajectories in volume over age (Fig. 5). Permutation testing confirmed that any significant differences in mean volume across the age bins, in selected subfields, were typically above the age of 60 with lower overall mean volume in APOE e3/e4 carriers compared to APOE e3/e3 carriers. This was most evident in the presubiculum head, subiculum body, CA4, GC-ML-DG and the hippocampal head, body and tail.

APOE e4/e4 status was associated with a significantly lower volume across subfields (Fig. 5). Different patterns emerged for the different subfields with regards to the age at which APOE e4/e4 carriers diverge from APOE e3/e3 and e3/e4 carriers, but in almost all subfields this divergence had occurred by age 65 and the decline was steeper from this age onwards.

This pattern was evident in para, pre and subiculum (ie the entire subiculum complex), the hippocampus amygdala transition area (HATA) as well as CA1 head, CA3, CA4 body and the molecular layers. Permutation testing confirmed these clear divergences in the curves for APOE e4/e4 carriers compared to both e3/e3 and e3/e4 carriers were significant. Those with the highest Alzheimer’s disease risk (i.e. older, homozygous e4 carriers) had reduced volumes in the same subfields that show reduced volume in mild cognitive impairment and Alzheimer’s disease, that is the subiculum, CA1, CA3 and CA4 (Wisse *et al.*, 2014). For some subfields, APOE e4/e4 status was associated with lower hippocampal volume from age 50 onwards, in particular the presubiculum and subiculum head, CA3 and CA4 body and the hippocampal head, tail and body. This points to the vulnerability of these subfields in APOE e4/e4 carriers from middle age. Overall, APOE status had differential interactions with age and APOE status across subfields, confirming non-linear effects that differ across the human hippocampus.

Importantly, control brain regions were also examined by APOE status across age. We did not find the same patterns observed in the hippocampus or its subfields when examining the ratio of volume loss relative to the rest of the grey matter (Supplementary Figs 2 and 3). Almost all control brain regions declined in volume with age in a linear way, with no clear effects of APOE status (Supplementary Figs 2 and 3).

### Differential effects of age, sex and APOE status on annualized cross-sectional subfield volume change

**Figure 6:**
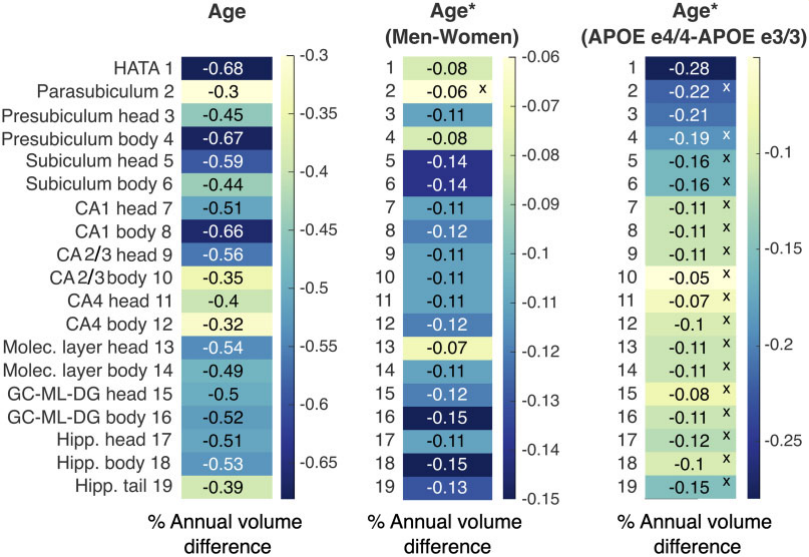
**Percent annual cross-sectional volume difference for each subfield for effects of age, age-by-sex, and age-by-APOE status interactions.** Normalized slopes were calculated in three separate linear models that included the effects of interest. Age had the strongest overall effects on annual volume change, followed by male sex. APOE e4/e4 status affected volume reduction beyond the effect of age only in the HATA and the presubiculum head.

To examine the differential effects of age, sex and APOE status on annualized cross-sectional subfield volumes in our data, the gradient of the main effect of age, the age-by-sex and the age-by-APOE status interactions were assessed (Fig. 6). Age had a significant effect on annualized volume across all subfields, with the greatest annualized reduction in volume observed in the HATA, presubiculum head, subiculum and CA1 body.

Significant age-related linear reduction in annualized cross-sectional volume was observed across all hippocampal subfields (Fig. 6), bringing together a number of the disparate previous findings by showing (cross-sectional) age effects across the hippocampus and its subfields that are above age-dependent global grey matter atrophy. Some of the highest annualized volume losses were observed in the presubiculum and subiculum, in line with findings which report presubiculum atrophy being associated with cognitive decline and low subiculum volume associated with long-term dementia risk (Evans *et al.*, 2018). Presubiculum and subiculum volume across ageing and APOE status may therefore be useful markers of future cognitive decline and may explain the relationships observed with both ageing and Alzheimer’s disease risk across studies.

The only subfields with a significant effect of APOE e4 homozygosity were the presubiculum head and the HATA, with around 0.21% and 0.28% annual reduction in volume in e4/e4 carriers relative to e3/e3 carriers. The absence of strong APOE e4 effects, in comparison to the sliding-window curves, reflects the linear model applied to estimated annualized volume change here.

Sex had a significant effect on all subfield volumes, except for the parasubiculum, though the overall effect was smaller in magnitude than the main effects of age. The sex effect indicates a greater reduction in annual cross-sectional volume change for men across all subfields (with the exception of the parasubiculum). The subfields with the greatest sex effects differ from those with the greatest age effects, confirming differential effects of age and sex on subfield volume.

## Discussion

Analysis of the largest population to date, consisting of over 36 653 healthy individuals, revealed significant interactions between age, sex and APOE status on the volume of the entire hippocampus, as well as differential effects on hippocampal subfields. Age, sex and APOE status are the strongest predictors of Alzheimer’s disease (Riedel *et al.*, 2016), warranting the search for sensitive MRI markers to detect brain changes that may indicate later vulnerability to disease (Barnes and Fox, 2014). The results presented here suggest hippocampal subfields have the potential to be important markers of known Alzheimer’s disease risk factors, in healthy individuals, when accounting for non-linear age effects and APOE and sex interactions.

A number of studies have shown differential effects of ageing across hippocampal subfields, with highly variable results as to which subfields are most vulnerable (Frisoni *et al.*, 2008; Wisse *et al.*, 2014; Carey *et al.*, 2019). CA1 is fairly consistent in its association with ageing. On the other hand, the subiculum has been shown to be both relatively spared in ageing, but implicated in Alzheimer’s disease-related atrophy (Wisse *et al.*, 2014; Daugherty *et al.*, 2016), and to be one of the subfields most affected by ageing (Kurth *et al.*, 2017; Malykhin *et al.*, 2017). The variable results reflect different cohort sizes, age ranges, MRI resolutions and subfield segmentation methods (Wisse *et al.*, 2014). We used data from a large population over a wide age range with high resolution, automated segmentation of hippocampal subfields. Therefore, our study benefits from the power to find age-related changes that may not be detectable in studies with smaller sample sizes. When examining linear reductions in age and annualized volume change, there was evidence of volume loss across all hippocampal subfields. This brings together a number of the disparate findings, by showing (cross-sectional) linear age effects across all subfields in a population of over 36 000 healthy ageing individuals.

The greatest annual volume losses were seen in the HATA, the presubiculum body, subiculum and CA1 body. The HATA is associated with information processing within the hippocampal-amygdala network and shows atrophy associated with cognitive decline and memory decline (Zheng *et al.*, 2018). Similarly, atrophy of the subicular complex, including presubiculum (Parker *et al.*, 2019), has been shown to be one of the earliest markers of Alzheimer’s disease and its atrophy and connectivity is associated with memory impairment (Carlesimo *et al.*, 2015; Hartopp *et al.*, 2018). Presubiculum and subiculum volume loss in 5035 dementia and stroke-free older adults has been shown to be associated with cognitive decline and low subiculum volume is associated with long-term dementia risk (Evans *et al.*, 2018). Presubiculum and subiculum volume across ageing may therefore be useful markers of future cognitive decline and may explain the relationships observed with both ageing and Alzheimer’s disease risk across studies. Finally, CA1 has been frequently implicated in ageing, and human autopsy studies show a particularly vulnerability to hypertension and ischaemia underlying volume loss (Mueller and Weiner, 2009; Shing *et al.*, 2011; Wisse *et al.*, 2014).

Examining linear trajectories of volume variation may obscure non-linear age effects in specific regions of the hippocampal complex. Using non-linear methods, we found volume loss was accelerated from age 65 in the hippocampus, and the presubiculum and subiculum, the HATA, CA1 head and CA3 and CA4 body subfields. The age effect is in line with a post-mortem study showing non-linear age-related cell loss in CA1-4 that is most evident after age 65 (Mani *et al.*, 1986) and an MRI study showing volume loss accelerated from age 65 to 85 in healthy older adults (Frisoni *et al.*, 2008).

The cross-sectional UK Biobank data presented here show a steady decline in whole hippocampal volume from age 50 to 65, followed by a much steeper decline after age 65 that is particularly pronounced in females. The risk of Alzheimer’s disease pathology expressing itself as clinical dementia is much greater in women than men (Barnes *et al.*, 2005). Women also have more global Alzheimer’s disease pathology (plaques and tangles observed at post-mortem), which has a stronger association with clinically diagnosed dementia than in men (Barnes *et al.*, 2005). Hippocampal volume decline might be a more prominent Alzheimer’s disease risk-related pathology in APOE e4 homozygous women than men. Our findings are in agreement with evidence that female e4 carriers with mild cognitive impairment have greater hippocampal volume loss and memory impairment than male e4 carriers (Fleisher *et al.*, 2005). The dramatic reduction in hippocampal volume in women over 65 may relate to the absence of ovarian hormones post-menopause, which are considered to have neuroprotective effects (Pertesi *et al.*, 2019; Rahman *et al.*, 2019; Vegeto *et al.*, 2020). While the average age of menopause is ∼50, because human life span has increased, women now spend a larger proportion of their life post-menopause (Morrison and Baxter, 2012). There is evidence of critical periods within which hormone replacement can mitigate the risk of Alzheimer’s disease (Henderson, 2006; Rocca *et al.*, 2011; Morrison and Baxter, 2012; Guo *et al.*, 2020). Our results present a trajectory of hippocampal volume that may inform critical periods before accelerated hippocampal volume loss creates an environment making those already at risk of Alzheimer’s disease (APOE e4/e4 older females) highly vulnerable to a cascade of neurodegenerative processes.

Here, we show strong age effects associated with Alzheimer’s disease risk factors in healthy individuals that indicates the selective vulnerability of subfields that may underlie cognitive deficits seen after the development of Alzheimer’s disease.

### Limitations

There have been remarkable gains in the spatial resolution of MRI and the ability to automatically segment subfields within the hippocampus. However, it should be acknowledged that these segmentations only approximate the gold standard, histology, in terms of precisely delineating subfields (de Flores *et al.*, 2015). UK Biobank MRI scan sequences were optimized to achieve the highest resolution feasible within a 35 min scan time, in order to scan 100 000 people. As a result, the structural scans were ∼1 mm resolution, known to be too low a resolution to distinguish the boundaries between CA and subiculum and the dentate gyrus at the cytoarchitectonic level. Therefore, the segmentations around these boundaries are driven by priors from the *exvivo* template data (Wisse *et al.*, 2014; Iglesias *et al.*, 2015). The design of our study minimizes the impact of these segmentation issues on the inferences we can draw. We did not compare raw volumes across groups (for example Alzheimer’s disease compared to healthy controls) but rather we compared differences between groups with age and APOE status, all of whom were segmented with the same method (see Supplementary material for an analysis of potential bias arising for the segmentation of the subfields and why this does not invalidate our results). The methods do make it possible to examine subfields at a population level, *in vivo*, which may aid biomarker discovery and understanding of the trajectory of hippocampal degeneration in ageing and neurological disease.

The study is also limited by the cross-sectional nature of the data analysed. Longitudinal studies can better capture changes in subfields over time by controlling for individual differences at baseline. Recent work by Nadal *et al.* (2020) showed that the annualized rate of atrophy in hippocampal subfields better predicted Alzheimer’s disease than baseline subfield volumes. However, we were not seeking to predict Alzheimer’s disease here, rather to track the trajectory of subfield volumes over age. An advantage of the current study is the ability to use population-level data and examine subfields across ageing—longitudinal studies are often limited to certain age groups due to the difficulty in following enough individuals of different age groups over time. Finally, the APOE e4/e4 group, given the low prevalence of the genotype, was the smallest group and interpretation of the effects of e4/e4 status should acknowledge that they will have the noisiest data.

## Conclusions

The results presented show that the subfields with the greatest sex effects differed from those with the strongest age or APOE effects. There were also interactions between risk factors that vary by subfield and likely account for the widely disparate findings in the current literature. These differential effects likely reflect the different functions, connections and classes of hippocampal synapses within the hippocampal complex (Morrison and Baxter, 2012). Although the neuronal loss and degeneration associated with Alzheimer’s disease are much more extensive, by examining the impact of major Alzheimer’s disease risk factors on subfield volumes we can determine the ageing environment that may make the onset of disease-related neurodegenerative processes more likely or faster. Hippocampal subfield volume may provide a sensitive index of vulnerability to later disease or cognitive decline.

## Supplementary material

Supplementary material is available at *Brain Communications* online.

## Supplementary Material

fcaa219_Supplementary_Data

## Abbreviations

APOE =: apolipoprotein E
CA1 =: Cornu ammonis 1
CA3 =: Cornu ammonis 3
CA4 =: Cornu ammonis 4
DG =: dentate gyrus
GC-ML-DG =: granule cell-molecular layer- dentate gyrus
KS =: Kolmogorov–Smirnov
IDP =: imaging derived phenotype
MRI =: magnetic resonance imaging
