# Supplementary material for: The human hippocampus and its subfield volumes across age, sex and APOE e4 status
Source: Brain Commun. 2020 Dec 19;3(1):fcaa219. doi: 10.1093/braincomms/fcaa219 (PMC7884607; doi:10.1093/braincomms/fcaa219)
Supplement: fcaa219_Supplementary_Data [file fcaa219_supplementary_data.pdf]

Supplementary material

Additional volume by age trajectories

Fig 1: Accelerated age-related volume loss in hippocampal subfields in APOE e4/e4 carriers relative to APOE e3/e4 and APOE e3/e3 carriers.

Fig 2: Ratio of control brain regions to the rest of the grey matter

Fig 3: Volume of control areas across age

Analysis of possible bias in subfield estimation

Fig 4: Scree plot of variance explained by principal components of the structural subfield volumes

Fig 5: Projection of principal components on coefficients.

Fig 6: Figure 5A reproduced with PCA including logarithmic terms

Additional volume by age trajectories

Figures 1-3.

Subfield and control area volumes, and control area ratio across age, splitting by APOE status. In all subfields, APOE e3/e3 and e3/e4 carriers had highly similar and overlapping trajectories in volume reductions over age. A sharp reduction in volume over age 65 in e4/4 carriers, is seen in the parasubiculum, the presubiculum and the subiculum head and body, and CA1 and CA3 head and CA4 body. Control areas show a largely linear decline with no clear APOE effect, with the exception of the amygdala.

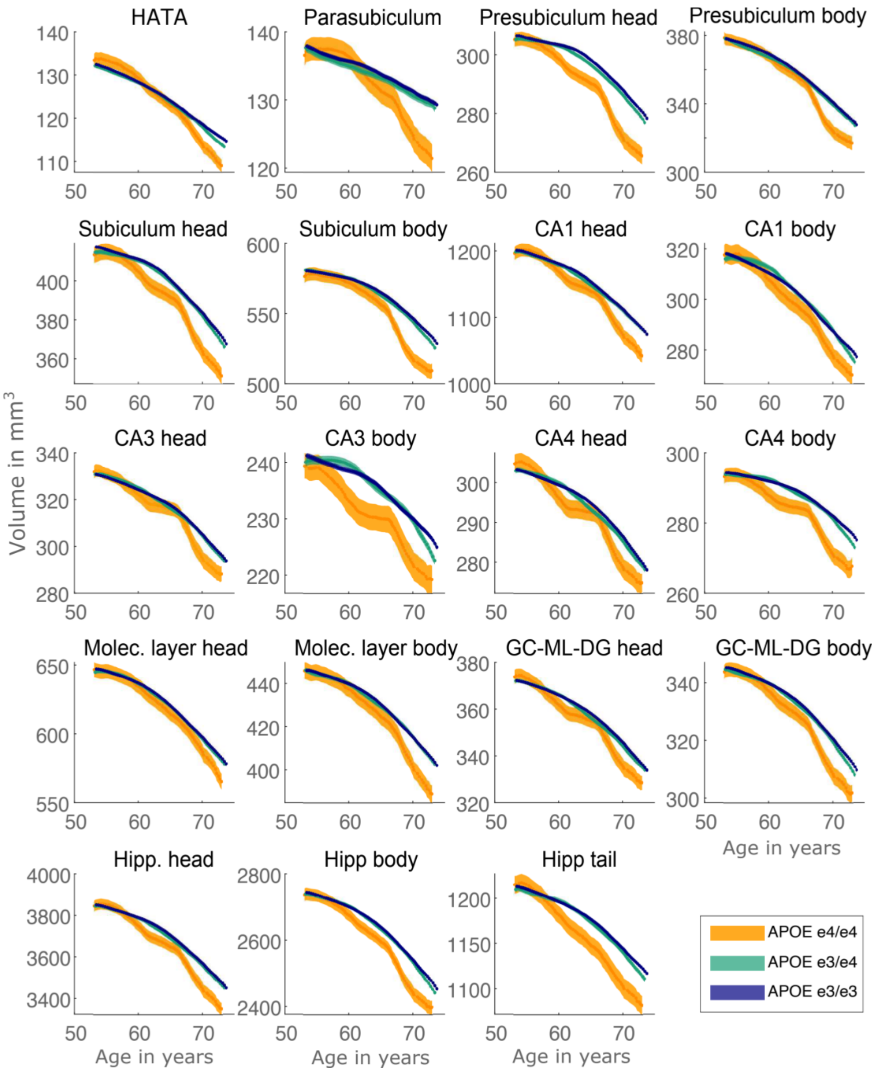

Fig 1: Accelerated age-related volume loss in hippocampal subfields in APOE e4/e4 carriers relative to APOE e3/e4 and APOE e3/e3 carriers.

A

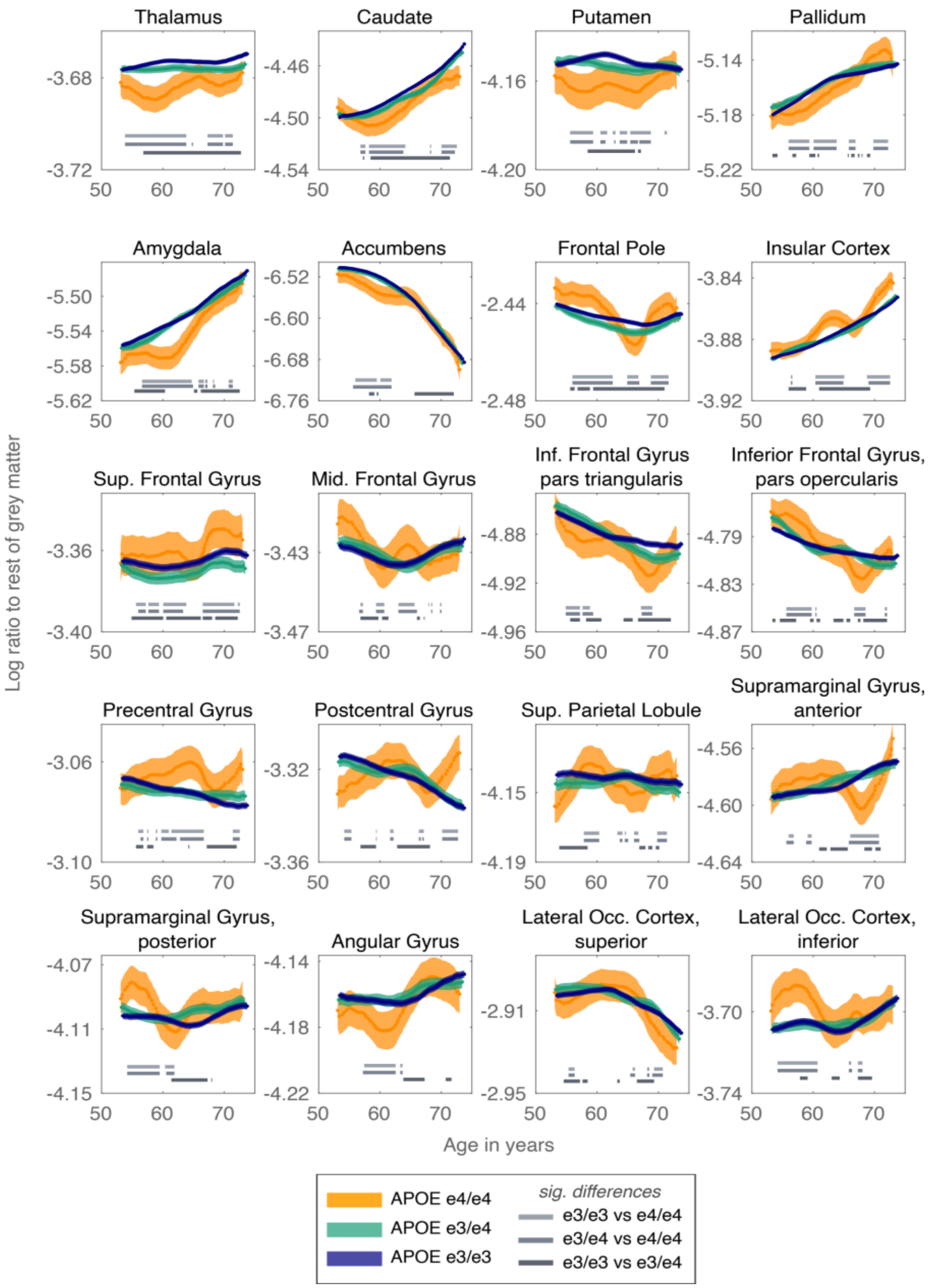

Fig 2: Ratio of control brain regions to the rest of the grey matter**. A**. Control brain regions areas do not show a clear APOE effect. Significant differences between the curves at p<.001 are shown per age window as grey horizontal bars. continued in part B

B

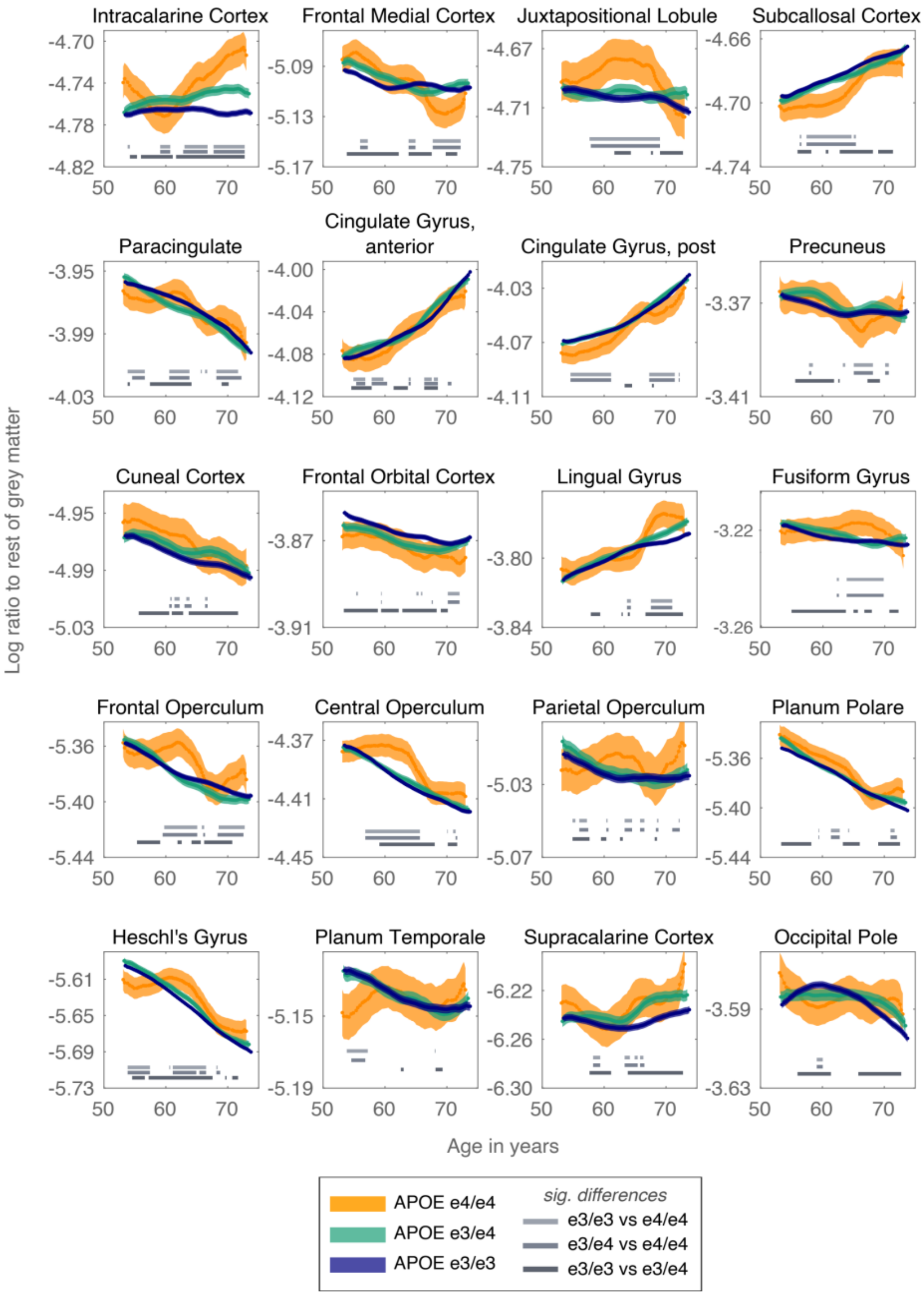

Fig 2: B. Further control brain regions areas do not show a clear APOE effect. Significant differences between the curves at p<.001 are shown per age window as grey horizontal bars.

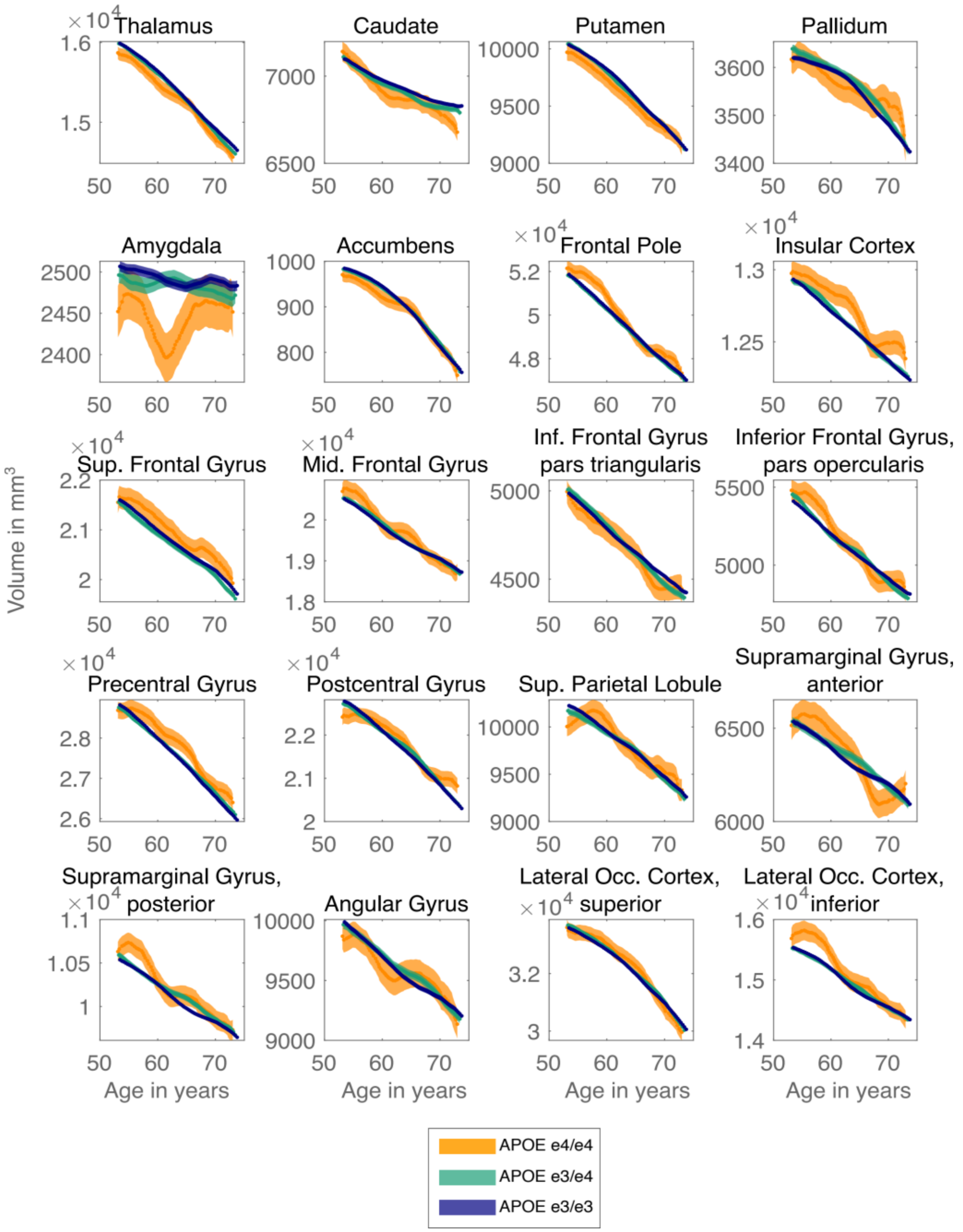

Fig 3: Volume of control areas across age**. A**. Volume of control brain areas declines linearly with age, with no clear APOE effect, with the exception of Amygdala. Continued in part B.

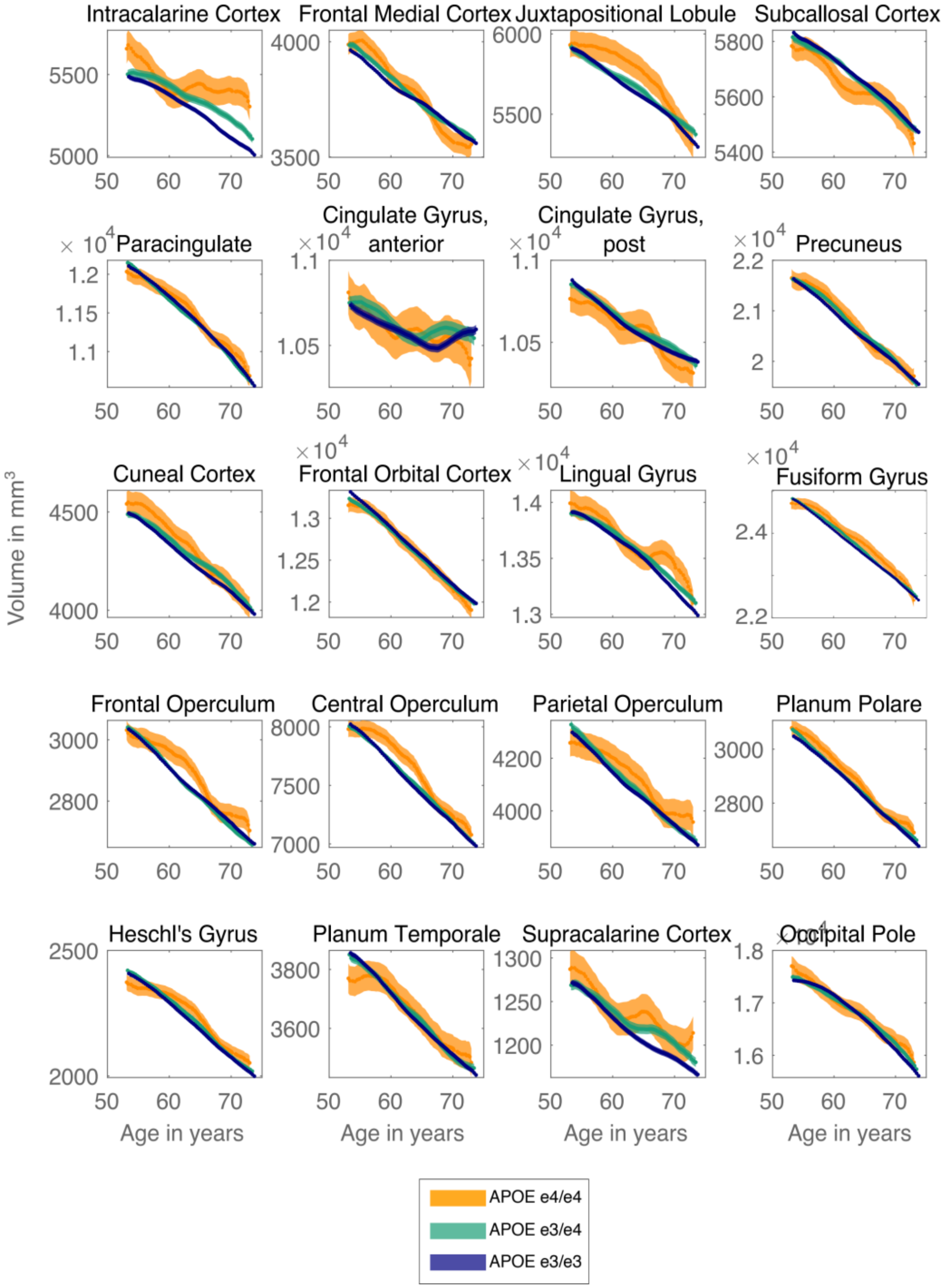

Fig 3: B. Volume of further control brain areas declines linearly with age, with no clear APOE effect.

Analysis of possible bias in subfield estimation

Figures 4-6.

A limitation of this study is that the resolution in structural scans from the UK Biobank is too low to distinguish the boundaries between CA and subiculum and the dentate gyrus at the cytoarchitectonic level. Therefore, the segmentations around these boundaries may be driven by priors from the ex-vivo template data (Iglesias et al., 2015) as explained in the discussion section. This would have been especially limiting if the raw volumes were being compared with other groups, or if we were interested in quantifying the amount of variability across people.

However, our analysis is designed specifically to compare differences in the estimates between our groups and with age. If the methods produce biases in the absolute volume measures, the bias should apply across all individuals. There are a number of types of bias that could be introduced, which we address below.

If the volume estimates are biased towards the priors, then for a given subregion, the volumes of different individuals would become more similar to each other. This would only weaken our results, because we are comparing how the pattern across regions differs between groups, who are estimated using the same method.

Similarly, if estimates primarily reflect total hippocampal volume, then this would also weaken our results regarding subfield differences. For a given individual, all subfields are biased in a particular direction, depending on that individual's total hippocampal volume, so that a null result (where subregions do not differ from each other) would not be interpretable. However, a significant difference between subregions, as shown in our analysis, would be hard to explain by the biases in Freesurfer's methods.

In addition, we addressed two main issues with Freesurfer that are noted in the literature (e.g. Wisse et al, 2014):

1. Systematic misattribution of areas from one subregion to another, due to the boundary priors
2. Misclassifying the axis on which atrophy occurs, e.g. CA1 atrophy misclassified as CA2/3 atrophy; De Flores et al. (2014) find this issue to be related to *proportional* underestimation of CA1.

Issue 1

If the prior affects the assignment of volumes to hippocampal subregions, this will occur in a systematic way, and will result in distortion of the axes of variance, in the space of possible hippocampal subregion volumes. In other words, the prior will result in systematic under- or over-estimation of volumes of particular subregions.

In turn, this will result in rotations of the structural covariance matrix of the subregions. One way to check our conclusions regarding selective APOE e4/e4 effects is to project the effects of age, sex*age and genotype*age (from Figure 6) onto principal components of the subregion volumes.

To obtain principal components, we used 21 subregions including temporal lobe and entorhinal cortex. Each region's volume was normalised to account for its overall size, and then equimax rotation was applied to optimally span the 7-dimensional subspace. The analysis revealed that 7 components explained 81% of the variance in volumes, illustrated in Figure 4. Each of the 7 components can be visualised as an 'atrophy pattern' over the subregions (Figure 5).

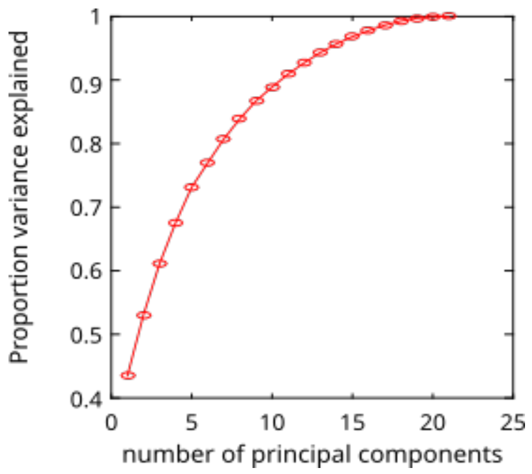

Fig 4: Scree plot of variance explained by principal components of the structural subfield volumes. Each component on the x-axis contributes a particular variation pattern across the subfields. The first 7 components account for 80% of variance across individuals.

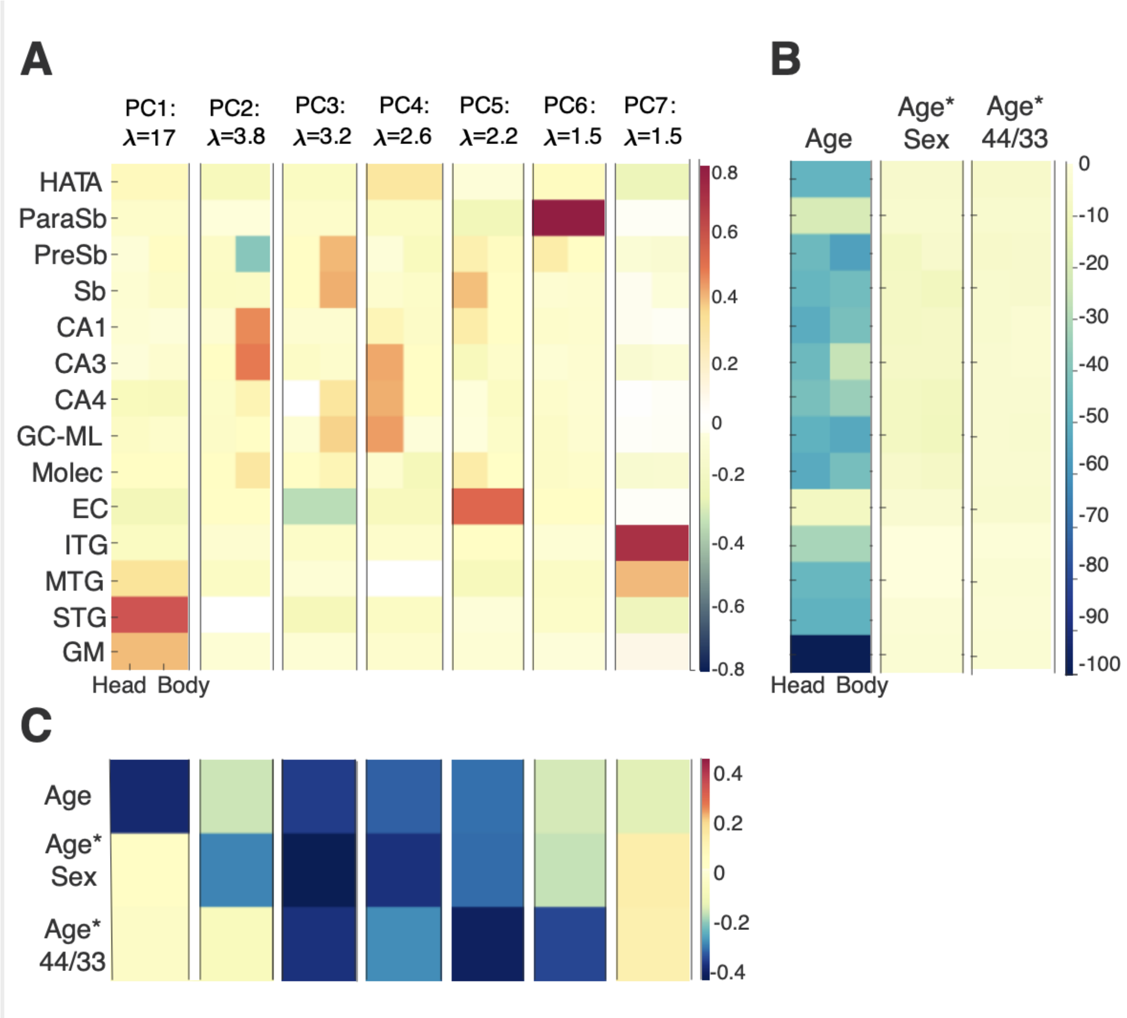

Figure 5: Projection of principal components on coefficients. **A**. The largest 7 principal components are illustrated as heatmaps across subfields. Each component has positive and negative effects on different subfields. The leftmost component is largest, as indicated by the eigenvalue (lambda) above. **B**. Normalised coefficients for age, sex, and the interaction of age and APOE status illustrated as heatmaps across subfields. **C**. Each factor's pattern of effects can be projected onto the 7 principal components. The decline due to age, the effect of sex on this decline, and the effect of genotype each have different loadings onto the components. This supports the conclusion that APOE has anatomically distinct effects on the hippocampus.

The three effects we assessed - age, age*sex, and age*allele - can be projected onto these components. Figure 5B shows a heatmap of these normalised coefficients similar to Figure 6, but now extended to match the volumes in Figure 5B. The amount that each of these effects loads onto these components, after normalising, is shown in the 5C.

Age loaded most strongly onto the first (largest) principal component -- which actually corresponds to non-hippocampal structures. For example, while there is a negative effect of age throughout, principal component 1 actually contributes a relative increase in entorhinal cortex (EC) volume (negative coeffcient * negative PC1 loading = positive contribution), while principal components 5 and 6 contribute a decrease in EC volume (negative coefficient * positive PC5 or PC6 loading = negative contribution). Thus, the components contribute in different directions, because they are chosen to explain as much variance as possible while keeping the patterns distinct from another.

Age also affected the other components which have hippocampal effects, but to a lesser extent. Age*sex loaded most strongly onto the third component which involved subiculum body most strongly, but also presubiculum, CA4, GC-ML and molecular layers. Age*ApoE genotype loaded most strongly onto component 5 that included parasubiculum, presubiculum and subiculum head and CA1, but also strongly affected EC.

The key aspect of this analysis is that this principal component loadings remain valid even if the priors bias the volumes or generate spurious correlations.

Issue 2

To partly address the issue of nonlinear biases in different regions, we performed the same analysis but included the logarithm of the volumes as additional variables in the PCA. Here, a multiplicative effect on volume will appear as an additive effect on the logarithm, and therefore a linear PCA that includes logarithmic terms can correct for biases whose sizes depend on volume. This analysis (42 variables) also required 7 components, and produced qualitatively highly similar results to the previous analysis without including the logarithms.

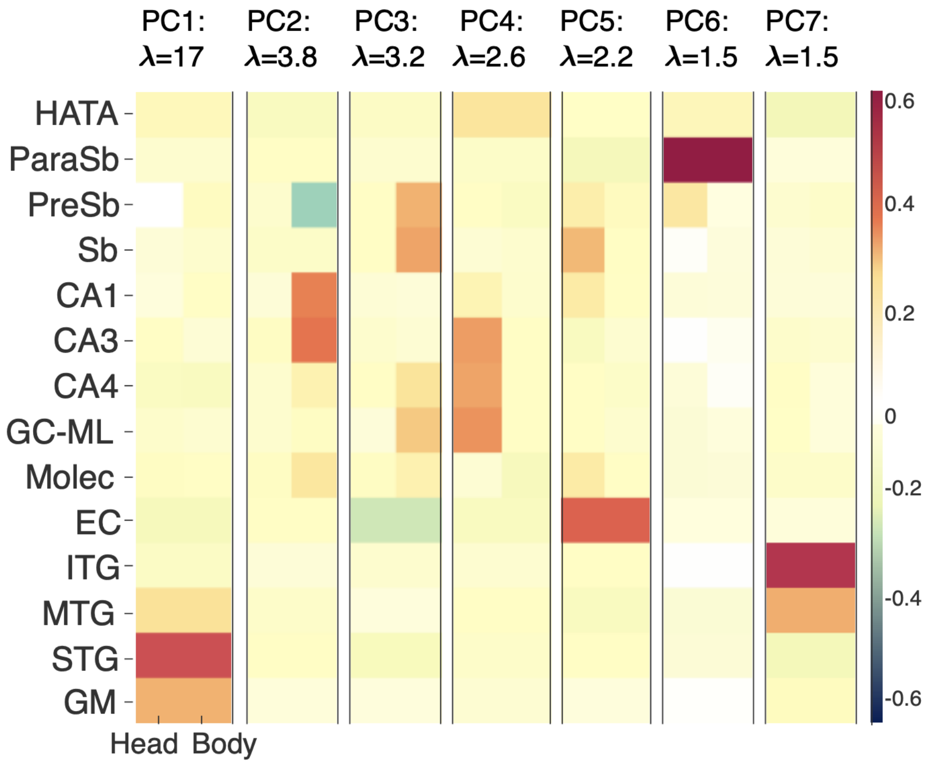

Figure 6: Figure 5A reproduced with PCA including logarithmic terms.

These analyses help confirm that APOE genotype results in distinct patterns of subregion volume, as determined by component-wise analysis. This pattern-wise analysis is more robust to the priors than a naive per-region analysis.

References

JE Iglesias, JC Augustinack, K Nguyen, CM Player, A Player, M Wright, N Roy, MP Frosch, AC McKee, LL Wald, B Fischl, Leemput K Van. A computational atlas of the hippocampal formation using ex vivo, ultra-high resolution MRI: Application to adaptive segmentation of in vivo MRI..*Neuroimage* **115**, 117-37 (2015).

LE Wisse, GJ Biessels, SM Heringa, HJ Kuijf, DH Koek, PR Luijten, MI Geerlings. Hippocampal subfield volumes at 7T in early Alzheimer's disease and normal aging.. *Neurobiol Aging* **35**, 2039-45 (2014)

de Flores, R, Joie, R, Chételat, G. Structural imaging of hippocampal subfields in healthy aging and Alzheimer's disease. *Neuroscience* **309,** 29-50 (2015).
